# A Practical Score for Prediction of Outcome After Cerebral Venous Thrombosis

**DOI:** 10.3389/fneur.2018.00882

**Published:** 2018-10-22

**Authors:** Miguel A. Barboza, Erwin Chiquete, Antonio Arauz, Marlon Merlos-Benitez, Alejandro Quiroz-Compeán, Fernando Barinagarrementería, Carlos Cantú-Brito

**Affiliations:** ^1^Stroke Clinic, Instituto Nacional de Neurología y Neurocirugía, Mexico City, Mexico; ^2^Neurosciences Department, Hospital Dr. Rafael A. Calderón Guardia, San José, Costa Rica; ^3^Department of Neurology, Instituto Nacional de Ciencias Medicas y Nutrición Salvador Zubirán, Mexico City, Mexico; ^4^Department of Neurology, Hospital Medica TEC100, Querétaro, Mexico

**Keywords:** cerebral venous thrombosis, mortality, scale, stroke, outcome

## Abstract

**Background:** Most patients with cerebral venous thrombosis (CVT) have independent survival in the short term. However, identification of high-risk individuals with an unfavorable outcome is a challenging task. We aimed to develop a CVT grading scale (CVT-GS) to aid in the short-term clinical decision-making.

**Methods:** We included 467 consecutive patients with CVT who were hospitalized from 1981 to 2015 in two third-level referral hospitals. Factors associated with 30-day mortality were selected with bivariate analyses to integrate a Cox proportional-hazards model to determine components of the final scoring. After the scale was configured, the prognostic performance was tested for prediction of short-term death or moderately impaired to death [modified Rankin scale (mRS) > 2]. CVT-GS was categorized as mild, moderate or severe for the prediction of 30-day fatality rate and a probability of mRS > 2.

**Results:** The 30-day case fatality rate was 9.0%. The CVT-GS (0–13 points; more points predicting poorer outcomes) was composed of parenchymal lesion size > 6 cm (3 points), bilateral Babinski signs (3 points), male sex (2 points), parenchymal hemorrhage (2 points), and level of consciousness (coma: 3 points, stupor: 2, somnolence: 1, and alert: 0). CVT was categorized as mild (0–2 points, 0.4% fatality rate), moderate (3–7 points, 9.9% fatality rate), or severe (8–13 points, 61.4% fatality rate). The CVT-GS had an accuracy of 91.6% for the prediction of 30-day mortality and 85.3% for mRS > 2.

**Conclusions:** CVT-GS is a practical clinical tool for prediction of outcome after CVT. This score may aid in clinical decision-making and could serve to stratify patients enrolled in clinical trials.

## Introduction

Cerebral venous thrombosis (CVT) is an uncommon cause of cerebrovascular disease accounting for < 1% of all acute strokes (1–3). Prognosis is usually good, with > 80% of patients attaining short-term independent survival (1, 4). Despite the highly favorable prognosis, identification of CVT patients with a possible unfavorable outcome can be challenging.

CVT mortality has been reported to range from 3 to 15% (1, 5), with the highest peak occurring in the acute phase of the disorder, particularly within the first 30 days (1, 6, 7). Predictors for an unfavorable outcome have been proposed from cohort studies including age, male sex, coma (5), encephalopathy syndrome (7), decreased level of consciousness, hemiparesis (8), seizures, intracerebral hemorrhage (5), involvement of the straight sinus, deep brain venous system thrombosis (7), venous infarction (8), cancer (5), central nervous system (CNS) infection (5), fever (7), and underlying hereditary thrombophilia (9). However, the relationship between these variables and the prediction of outcome is not widely established. Moreover, based on hospital registries, there are some inconsistencies in the distribution of CVT causes, identified risk factors and outcome rates.

Some risk scoring models have been developed to predict prognosis at the single patient level (6, 10–12). These models are mainly aimed to identify patients in need of intensive care, but there have been some limitations related to validity (especially low specificity), possibly due to the fact that none of them have been developed exclusively for mortality prediction. The aim of the present study was to establish risk predictors for bad functional outcome and death in acute CVT patients, and to provide a classification system that allows prognosis assessment.

## Methods

### Study design and population

This is a retrospective study on a systematic database prospectively arranged about 467 consecutive Mexican mestizo patients with confirmed CVT, who were hospitalized from November 1981 to April 2015 in two tertiary care teaching centers in Mexico City (the National Institute of Neurology and Neurosurgery and the National Institute of Medical Sciences and Nutrition). Our standardized database systematically collects demographic and clinical data, imaging including computed tomography (CT) scan, magnetic resonance imaging (MRI) or digital subtraction angiography, laboratory studies, in-hospital, and outpatient follow-up information data, as well as functional status.

The primary outcome of our study was to derive a practical yet robust severity classification system for death and bad functional outcome in CVT patients in the acute setting (30 days); the secondary outcome was to compare its clinical performance with a previously validated risk score derived from the ISCVT study and the VENOPORT registry (the ISCVT-RS system), as the reference scale (6).

Patients fulfilling inclusion criteria should be: confirmed CVT, through brain imaging of occluded sinus and/or cortical veins or autopsy confirmed diagnosis; adult CVT cases (> 18 years-old), and patients with substantial clinical and functional status information from their medical records that could be extracted for the analysis.

We excluded patients with unconfirmed diagnosis of CVT on imaging tests and records with incomplete clinical information and functional status at 30 days of follow up, also CVT cases associated with central nervous system infections, or parameningeal infections. A subset of patients included in this registry has been followed for more than 25 years. The Institutional Review Board at each institution approved the CVT registry and the present analysis. Three investigators (MAB, EC, and CCB) assessed the uniformity of the data included in the database from both centers.

### Data collection

The main data requested from each patient included date of diagnosis, age, gender, family or personal history of thrombophilia, recent obstetric history (pregnancy, puerperium, abortion, preeclampsia/eclampsia, and septic complications during pregnancy or puerperium), personal history of vascular risk factors, extracranial venous thrombosis, oral contraceptives or hormonal replacement therapy use and history of parameningeal CNS infections. Clinical information at hospital arrival included consciousness and mental status, seizures at onset, motor or sensitive deficit, headache, visual and speech disturbances, intracranial hypertension syndrome, fever, meningeal syndrome, elapsed time since first signs and symptoms to hospital admission, hemoglobin and hematocrit. In-hospital information included acute neurological worsening during hospitalization, mechanical ventilation support, type of medical intervention (anticoagulation, antiplatelet therapy or none), decompressive craniotomy, number of in-hospital days and functional status. Neuroimaging information included the type of study, direct signs of sinus/vein thrombosis (empty delta sign, hyperdensity or hyperintensity of cerebral sinuses), affected veins or sinuses, localization, categorization of involved venous system (superficial, deep or both), parenchymal lesions (venous infarction, hemorrhagic transformation, and/or hematoma), and subarachnoid hemorrhage. Follow-up information included the modified Rankin score (mRs) at discharge, 30 and 90 days of outpatient follow-up, early (≤ 30 days); CVT-related death, long-term medical treatment (oral anticoagulation, antiplatelet therapy, or none), and neurological sequelae status at last follow-up. All data were thoroughly revised for consistency. The functional outcome was evaluated with the mRS dichotomized as good (mRs 0–2) and bad (mRs > 2); for the purpose of the present analysis, only information related to the functional status according to mRs on the first 30 days was included.

Diagnosis delay refers to the lapse between symptoms onset and CVT diagnosis.

Malignancy variable was determined both as the patient's past history of a neoplasm and diagnosis reached prospectively during hospitalization.

### Definitions of critical variables

Seizures were defined as focal or generalized involuntary motor convulsion categorized as either focal, with generalization (bilateral activity) or status epilepticus; all patients included in this variable should have clinical and subsequent standard electroencephalogram evaluation, according to institutional protocols. Coma was defined as persistent disturbance of consciousness, the patient being non-alert and non-arousable with any type of stimulus, and a Glasgow coma scale (GCS) scoring of < 9 points measured at admission. Stupor was defined as a patient with GCS < 9 who was momentarily arousable with a noxious stimulus (6). Meningeal or radicular irritation signs were recorded when neck stiffness, Kernig and/or Brudzinski signs were detected. CVT topography was categorized according to the individual affected veins and gross location (i.e., superficial, isolated deep, or mixed). Lesion size in venous/hemorrhagic infarction or focal edema is not clearly established in previous CVT articles, therefore we developed a direct measurement of the highest diameter (centimeters) of the venous infarction or hemorrhage, traced on the brain computed tomography (CT) or magnetic resonance (MR) on the slice with the highest area of parenchymal lesion, and the subscale system (to define the cut-off value with the highest sensitivity) was established according to the performance from all the diameter sizes in a ROC for the composite of death and bad functional outcome.

Mixed venous systems thrombosis was defined as the combination of any of superficial (superior longitudinal sinus, lateral and sigmoid sinus, and/or jugular vein) and deep (Rosenthal's basal vein, inferior longitudinal sinus, straight sinus venous, thalamus-striate vein) venous thrombosis.

### Statistical analysis

Parametric continuous variables are expressed as geometric means with standard deviation (SD), or the minimum and maximum. Non-parametric continuous variables are expressed as medians with interquartile range (IQR). Categorical variables are expressed as percentages. A first step of bivariate analyses was performed to identify variables associated with 30-day mortality, by Pearson chi-square or Fisher exact tests, as appropriate. Continuous variables such as age and lesion size were dichotomized to transform them into nominal variables, using the median value or its nearest integer. To find independent predictors of 30-day mortality, multivariate analyses were constructed by forward stepwise logistic regression on potential predictors as first detected after hospital arrival. Input variables were those that were found to be significantly associated with mortality in the bivariate analyses, with a significance level set to *p* < 0.10 (only to integrate the multivariable analysis). The continuous variables of age and lesion size first entered the model as natural integers. Adjusted odds ratios (OR) with 95% confidence intervals (CI) are provided. The fitness of the model was evaluated using the Hosmer-Lemeshow test for goodness of fit, which was considered as reliable when *p* > 0.2. To further assess the reliability of the effect size of every independent predictor, a Cox proportional hazards model was also constructed, considering the time variable as the elapsed period between hospital arrival and discharge. The preliminary prediction models were considered reliable when the hazard ratio (HR) of each independent variable approximated the corresponding OR provided by the logistic regression analysis with a difference of no more than 0.25 between the effect sizes. Once independent predictors of 30-day mortality were identified (those with ORs not crossing the unit and as a consequence with *p* < 0.05), continuous variables were dichotomized in order to test the cut-offs with the best prognostic performance by adjusting several preliminary scales with different age and lesion size cut-offs into a receiver operating characteristic curve (ROC). The scale with the greatest area under the ROC (AUC) was selected as the final grading scale. The CVT grading scale (CVT-GS) was designed by allotting points to each independent variable based on their effect size as follows: the reference unit was the variable with the lowest OR, which was assigned the lowest score (i.e., 1 point), and the ORs of the rest of the variables were divided by that of the reference unit. The resultant quotients were rounded up to the nearest integer. Statistical comparisons or interactions with p < 0.05 were considered statistically significant.

Analyses of prognostic performance were carried out for both the CVT-GS and International Study of CVT risk score (11), (ISCVT-RS, 0-9 points, more points meaning a worse outcome) as the reference system. Spearman's rank correlation (*r*) and determination (*r*^2^) coefficients were calculated to estimate the amount of variance in acute outcome explained by both scales. The sensitivity, specificity, Youden index, positive predictive value (PPV), negative predictive value (NPV), and likelihood ratios for positive and negative test results (LR+ and LR–, respectively) were calculated considering the discrete value of each scale with the greatest Youden index (4 points for CVT-GS, and 3 for ISCVT-RS). The prognostic accuracy was estimated by calculating the AUC, under continuous non-parametric assumptions. All parameters of the prognostic appraisal (validity and reliability) are expressed as percentages with the corresponding 95% CI. The CVT-GS was validated internally by using the bootstrap method in the original derivation dataset by sampling with replacement for 100 iterations. Each bootstrap sample received accuracy analyses by calculation of the AUC in order to estimate the degree to which the predictive accuracy would be expected to deteriorate when applied to an independent external sample with comparable characteristics. The CVT-GS scoring was divided into tertiles to allow for the classification system of mild, moderate and severe. Kaplan-Meier survival analyses were also performed with the clinical CVT-GS cut-offs.

## Results

In all, 507 records of patients with CVT were identified as potential study subjects. Among them, 40 (7.8%) were excluded because they did not fulfill the selection criteria (17 patients had acute or chronic CNS infections or septic cavernous sinus thrombosis, 7 patients had unconfirmed CVT, 5 patients had incomplete clinical information at follow-up, 5 patients had missing records, and 6 patients were lost at follow-up). Therefore, 467 patients composed the final study sample: 381 (81.6%) women and 86 (18.4%) men with a median age of 29 years (IQR 22 to 38 years; Table 1). The mean duration of the hospital stay was 14.7 ± 10.7 days. CVT related to obstetric conditions was present in 224 (47.9%) of the women. The 30- and 90-day case fatality rates were 8.7 and 9.2%, respectively. A good 30-day outcome occurred in 359 (76.9%) patients.

**Table 1:** Clinical and demographic characteristics of the 467 patients included in this study.

**Variable**	**All patients**	**30-day status**		*P*-value^*^
	**(*n* = 467)**	**Alive (*n* = 425)**	**Death (*n* = 42)**	
Age, mean (SD), y	31.3 (12.4)	30.9 (12.3)	34.2 (13.6)	0.14
Women, *n* (%)	380 (81.4)	347 (81.6)	33 (78.6)	0.62
Men, *n* (%)	87 (18.6)	78 (18.4)	9 (21.4)	0.62
Diagnosis delay, mean (SD), days	17.9 (35.4)	18.6 (36.8)	10.2 (10.7)	0.11
**RISK FACTORS**, ***n*** **(%)**				
Smoking	61 (13.1)	57 (13.4)	4 (9.5)	0.47
Malignancy	12 (2.6)	8 (1.9)	4 (9.5)	0.003
Thrombophilia	25 (5.4)	24 (5.6)	1 (4.0)	0.37
Pregnancy and puerperium	216 (46.4)	198 (46.7)	18 (42.9)	0.63
**CLINICAL CHARACTERISTICS**, ***n*** **(%)**				
Headache	396 (84.8)	363 (85.4)	33 (78.6)	0.23
Level of consciousness				< 0.001
Awake and alert	293 (62.7)	289 (68.0)	4 (9.5)	
Somnolence	133 (28.5)	114 (26.8)	19 (45.2)	
Stupor/coma	41 (8.8)	22 (5.2)	19 (45.2)	
Focal seizures	60 (12.8)	54 (12.7)	6 (14.3)	0.77
Bilateral Babinski signs	93 (19.9)	64 (15.1)	29 (69.0)	< 0.001
Papilledema	183 (39.2)	167 (39.3)	16 (38.1)	0.88
**NEUROIMAGING**, ***n*** **(%)**				
Venous infarction	167 (35.8)	161 (37.9)	168 (36.0)	0.006
Bilateral parenchymal hemorrhage	129 (27.6)	109 (25.6)	20 (47.6)	0.002
Parenchymal hemorrhage	184 (39.4)	152 (35.8)	32 (76.2)	< 0.001
Parenchymal lesion size >6 cm	84 (18.0)	55 (12.9)	29 (69.0)	< 0.001
Cerebral venous system involvement				0.002
Superficial	365 (78.2)	340 (80.0)	25 (59.5)	
Deep (isolated)	17 (3.6)	16 (3.8)	1 (2.4)	
Mixed (superficial and deep location)	85 (18.2)	69 (16.2)	16 (38.1)	
**TREATMENT**, ***n*** **(%)**				
Anticoagulant therapy	222 (47.5)	205 (48.2)	17 (40.5)	0.34
Surgery (hemicraniectomy)	15 (3.2)	10 (2.4)	5 (11.9)	0.001

*SD, standard deviation*.

* *P-value for differences between men and women; Pearson chi-square, Fisher exact test, Student t-test or Mann-Whitney U-test, as appropriate*.

The resultant variables associated with a high mortality risk were male gender, level of consciousness (stratified as awake and alert, somnolence, stupor and coma), bilateral Babinski sign, parenchymal hemorrhage and parenchymal lesion size > 6 cm. This model proved to be the best after retesting in a multivariate adjusted model (Table 2). Age did not show a strong contribution to the final model.

**Table 2:** Cox proportional hazards models for prediction of mortality and mRS >2 at 30 days after CVT.

**Variable**	**HR**	**95% CI**	***P*-value**
**30-DAY MORTALITY**			
Parenchymal lesion size > 6 cm	3.144	1.493–6.622	0.003
Bilateral Babinski signs	2.677	1.184–6.057	0.018
Male gender	2.277	1.030–5.036	0.042
Parenchymal hemorrhage	2.161	1.027–4.548	0.043
Level of consciousness^*^	1.923	1.334–2.773	< 0.001
**30-DAY mRS** > **2**			
Parenchymal lesion size > 6 cm	1.278	1.109–1.471	0.001
Bilateral Babinski signs	1.287	1.099–1.508	0.002
Male gender	1.366	1.076–1.734	0.010
Parenchymal hemorrhage	1.449	1.193–1.760	0.030
Level of consciousness^*^	1.618	1.260–2.079	< 0.001

*CI, confidence interval; HR, hazard ratio*.

* *Models adjusted for patients' age, diagnosis delay, etiology, clinical features, venous system localization (superficial, deep or mixed), and treatment (anticoagulation, surgery). Hazard ratios for each of the following consciousness levels: awake and alert, somnolence, stupor, and coma. Only variables significantly associated with the two study outcomes are included in the final steps of the models*.

**Figure 1:**
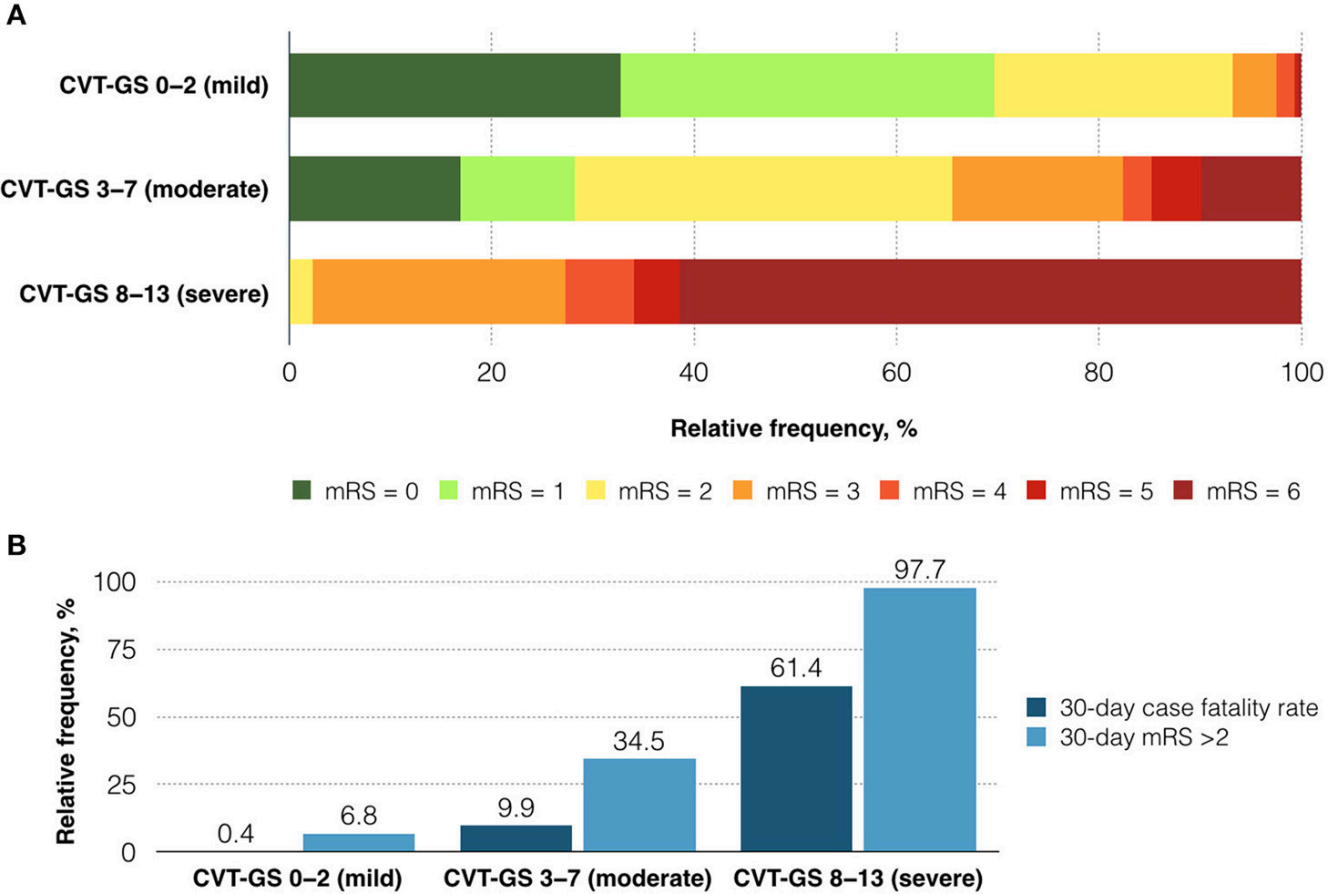
mRS at 30 days after CVT according to CVT-GS severity category **(A)**. Thirty-day case fatality rate and mRS > 2 according to the CVT-GS severity category **(B)**.

**Figure 2:**
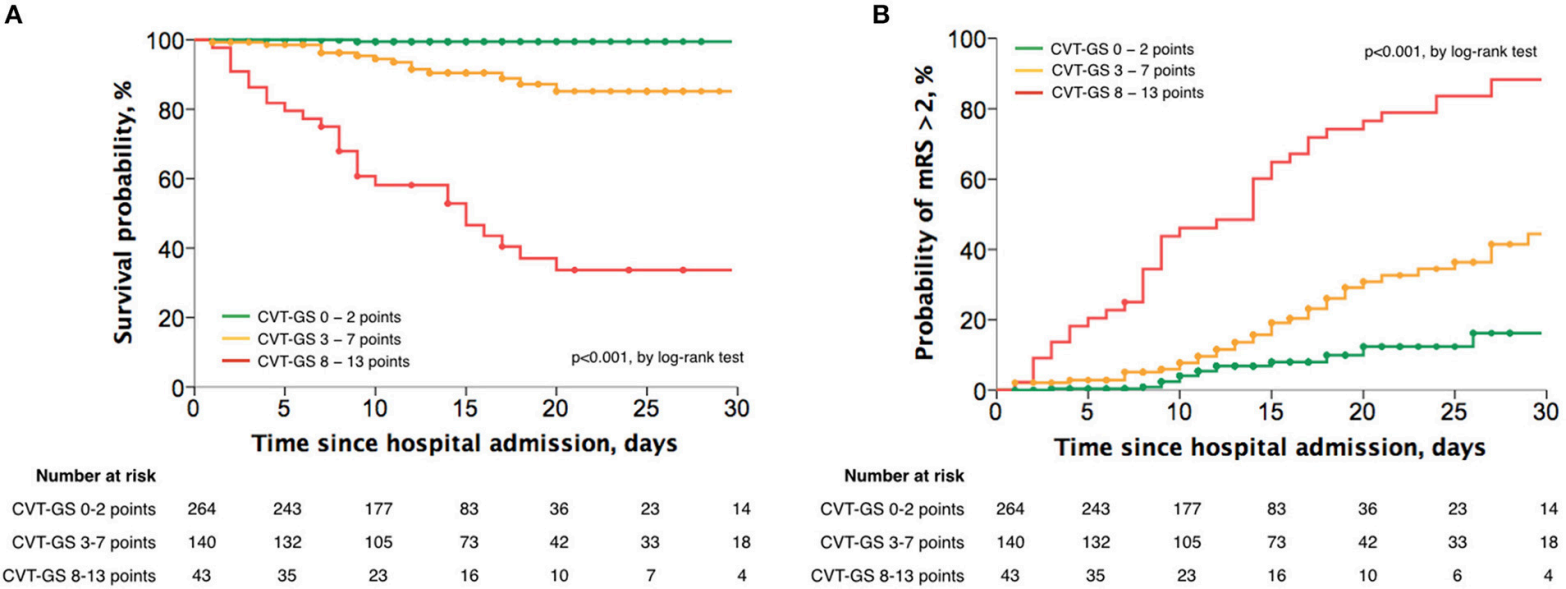
Actuarial analyses with the Kaplan-Meier method for the survival probability **(A)** and the probability of attaining a mRS > 2 **(B)** at 30 days after CVT, according to the CVT-GS categories.

With the final risk model selected, points were assigned to each variable according to the size effect in the prediction of death from the adjusted HR. The 30-day case fatality rate was 9.0%. The composed CVT-GS had a range from 0 to 13 points, with more points predicting poorer outcomes: parenchymal lesion size > 6 cm (3 points), bilateral Babinski signs (3 points), male sex (2 points), parenchymal hemorrhage (2 points), and level of consciousness (coma: 3 points, stupor: 2, somnolence: 1, and alert: 0; Table 3). As a consequence, CVT was categorized into mild (0–2 points), moderate (3–7 points), and severe (8–13 points). CVT-GS had an accuracy of 91.6% in the prediction of 30-day mortality, and 85·3% for prediction of mRS > 2. A mild CVT implied 1.1% mortality (HR: 0.1; 95% CI: 0.04–0.20; *P* < 0.001), moderate CVT indicated 19.6% mortality (HR: 2.4; 95% CI: 1.30–4.51; *P* = 0.005), and severe CVT suggested 61.4% mortality (HR: 12.4; 95% CI: 6.61–23.32; *P* < 0.001; Figure 1). Relative frequency from the functional clinical outcome categorized according to each CVT-GS category can be seen in Figure 2.

**Table 3:** Critical appraisal on the prediction performance of ISCVT-RS (reference system) and CVT-GS (proposed system).

**Prognostic variable**	**ISCVT-RS (Risk score range: 0–9 points)**	**CVT-GS (Risk score range: 0–13 points)**
**RISK SCORING**		
Malignancy	2	–
Coma	2	–
Thrombosis of the deep venous system	2	–
Mental status disturbance	1	–
Male gender	1	–
Parenchymal hemorrhage	1	–
**RISK SCORING**		
Parenchymal lesion size > 6 cm	–	3
Bilateral Babinski signs	–	3
Male gender	–	2
Parenchymal hemorrhage	–	2
Level of consciousness		
Awake and alert	–	0
Somnolence	–	1
Stupor	–	2
Coma	–	3
**30-DAY MORTALITY**^‡^		
Sensitivity (95% CI)	0.476 (0.334–0.623)	0.714 (0.564–0.828)
Specificity (95% CI)	0.887 (0.853–0.914)	0.929 (0.901–0.95)
c-statistic (95% CI)	0.913 (0.866–0.959)	0.786 (0.715–0.856)
J-statistic (95% CI)	0.501 (0.359–0.624)	0.620 (0.481–0.727)
PPV (95% CI)	0.294 (0.199–0.411)	0.500 (0.377–0.623)
NPV (95% CI)	0.945 (0.918–0.963)	0.971 (0.949–0.983)
LR+ (95% CI)	4.216 (2.786–6.38)	10.119 (6.821–15.012)
LR– (95% CI)	0.591 (0.442–0.789)	0.307 (0.19–0.496)
**30-DAY mRS** >**2**^‡^		
Sensitivity (95% CI)	0.667 (0.564–0.755)	0.700 (0.599–0.785)
Specificity (95% CI)	0.814 (0.757–0.860)	0.867 (0.817–0.905)
c-statistic (95% CI)	0.828 (0.778–0.877)	0.707 (0.651–0.763)
J-statistic (95% CI)	0.498 (0.319–0.744)	0.590 (0.461–0.701)
PPV (95% CI)	0.594 (0.497–0.685)	0.677 (0.577–0.764)
NPV (95% CI)	0.856 (0.802–0.898)	0.879 (0.830–0.915)
LR+ (95% CI)	3.580 (2.620–4.890)	5.270 (3.680–7.560)
LR– (95% CI)	0.410 (0.300–0.550)	0.350 (0.250–0.480)

*CI, confidence interval; CVT, cerebral venous thrombosis; HR, hazard ratio*.

‡ *Prognostic performance with the actual median cut-off of 3 points for ISCVT-RS and of 6 points for CVT-GS. ISCVT-RS has a possible scoring range from 0 to 9 points; however, neither in the derivation and validation cohorts nor in the present study did a single patient score > 6 points, and hence, the actual median cut-off score for ISCVT-RS is 3 instead of 5, as can be expected by the 0 to 9 score range*.

**Figure 3:**
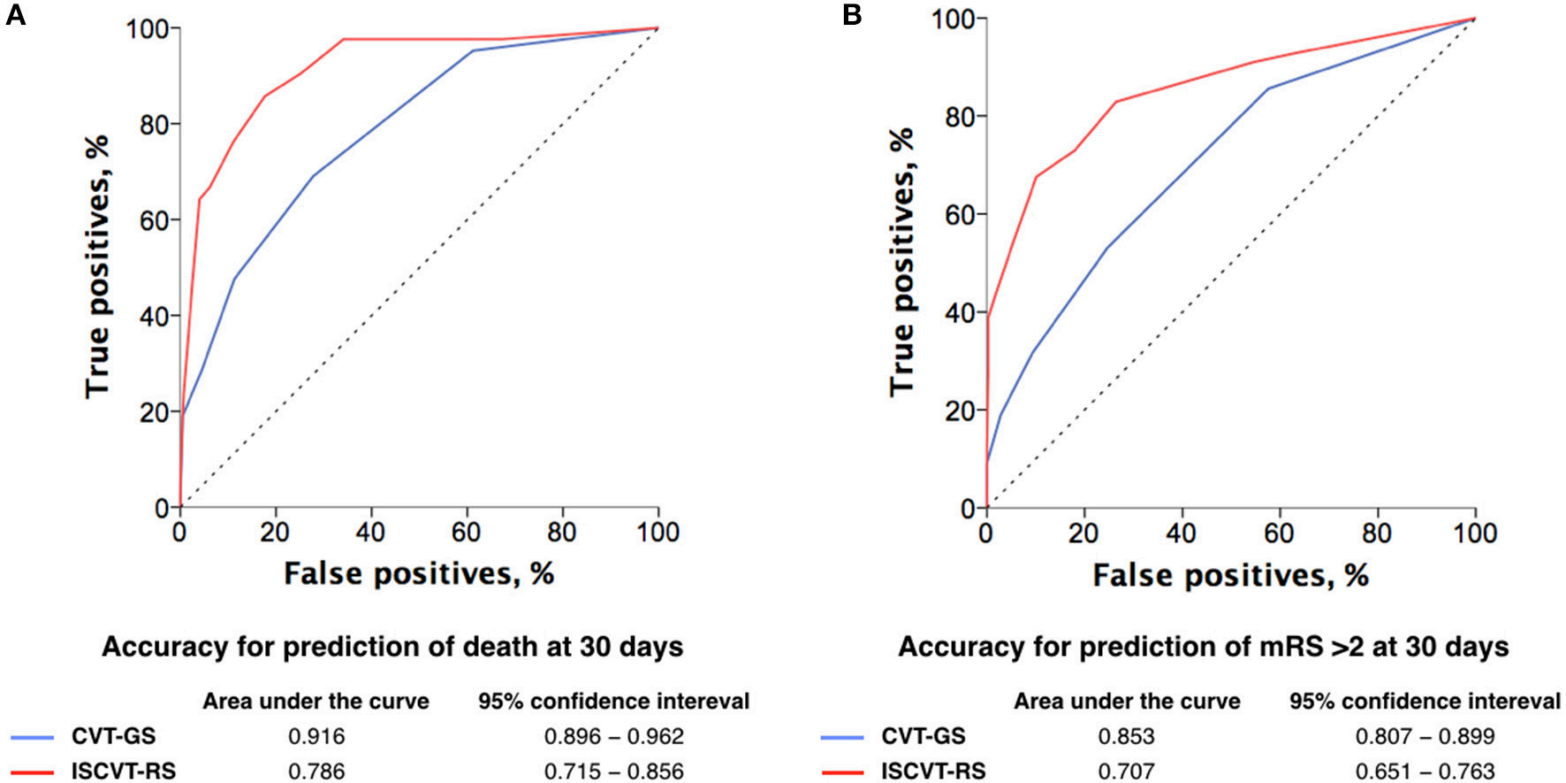
ROC curves for CVT-GS and ISCVT-RS accuracy analysis for the prediction of death **(A)** and mRS > 2 **(B)** at 30 days after CVT.

**Figure 4:**
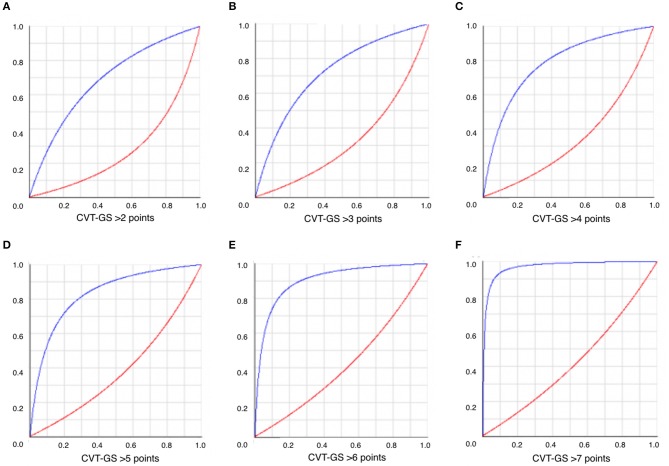
Positive (blue lines) and negative (red lines) post-test probability of mRS > 2 at 30 days according to different CVT-GS cut-off **(A-F)**.

As compared with ISCVT-RS (with a testing cut-off = 3 points), CVT-GS (testing cut-off = 4 points) was globally better in predicting 30-day mortality (HR: 4.9; 95% CI: 2.66–8.96 vs. HR; 15.1; 95% CI: 7.68–29.42, respectively), as shown by a significantly greater accuracy (Figure 3) and Youden's index (Table 3). Positive and negative post-test probability presented a classical inverting pattern with the increase in CVT-GS cut-offs scoring (Figure 4), as can be expected for a clinical grading scale.

## Discussion

We have derived a grading system for the severity of CVT based on a systematic approach. Our model showed improvement over the most important previous reference (ISCVT-RS), considering both the sample size and the methodology of derivation. Some previous models have used a pragmatic arbitrary approach to select prediction variables (6, 10–12), which may explain some of the observed limitations in validity and reliability. Indeed, ISCVT-RS showed a better prognostic performance in our dataset, as compared with that observed in the original derivation sample (6). As shown in our analysis, a probabilistic approach may yield superior conclusions than that of an arbitrary system based on clinical expertise alone. Some variables are related with outcome as epiphenomena that run in parallel with other truly independent factors. A probabilistic systematic approach may offer greater reliability when designing a prognostic scale or severity classification system.

Some risk factors identified in different cohorts have not been validated as independent predictors of outcome in further studies (6–13). This could be explained by demographic, genetic, and epidemiological differences across the sample populations. Therefore, to establish the local risk behavior and to assess the performance of a prognostic system it is very important to increase the external validity of the proposed prognostic tools. Our model exhibits some benefits in clinical practice. All predictive risk variables are easy to achieve in many hospital scenarios with routine clinical and neuroimaging evaluations performed upon hospital arrival. The highest risk of mortality and bad outcome has been observed within the short-term setting (5, 13–19), which becomes a critical period to define therapeutic actions for this group of patients. The CVT-GS can be used to estimate the risk of early mortality or a clinically unacceptable bad outcome in CVT patients (mRS > 2). The ISCVT-RS as the reference comparative system is considered much better at predicting good outcomes and may be less accurate for identifying patients with expected poor outcome (6). Therefore, our scale achieves its best performance in predicting acute fatality. Another reason to establish our primary goal as the evaluation of functional bad outcome in the acute period is related to the lack of statistical significance in the mid- and long-term functional outcome (3 and 6 months, respectively), with a tendency for a good prognosis (mRS 0–2) in those who overcome the 30-day initial period (13, 15–24). However, our score model must be evaluated in another prospective study to test its performance in these follow-up periods as well.

The optimal medical treatment in patients with a bad functional condition in the acute phase is still uncertain, even though anticoagulation remains as the best recommendation for CVT medical management (1), and in severe cases, surgical decompressive approaches, endovascular (25), or thrombolytic therapy (26, 27) have been used in several case series, especially in patients with rapidly progressive clinical worsening. CVT-GS allows for establishment of risk groups, which may help in clinical decision-making, and as a consequence, this model can be used to stratify patients with high risk of death, to identify patients who have an indication of the need for more aggressive treatment and to determine those to be ideally recruited into interventional trials.

This study also confirmed that most patients with CVT have a benign prognosis; the fatality rates at 30 and 90 days were 8.7 and 9.2%, respectively, and most surviving patients recovered completely or had only mild functional outcomes. Even though the individual time course in patients with CVT is highly variable, our results suggest that the risk of death is increased during the acute phase and that there is a significantly good functional outcome in those who overcome the initial 30-day period.

This study does have some limitations that should be taken into account for the correct interpretation of data. First, no external validity with a different population dataset was performed to assess the clinical performance in different scenarios. This is an important factor that may limit the adoption of this and previous risk classification systems. Also, even though most of the variance in outcomes occurs within the first 30 days after hospital admission, it may be perceived that the current prediction models, including the CVT-GS, have not been adequately tested in the long term. Another limitation may be the elapsed time since the inclusion of the first patients in the present registry. Although we did not observe a significant difference in prognostic performance of the CVT-GS before and after 1992 (the year that the first venous MRI was performed in our patients), important advances have been reached regarding the diagnosis of CVT that currently allow for an earlier diagnosis than decades ago. Nevertheless, no significant advances in specific therapies have changed the fate of CVT patients (3).

## Conclusion

The CVT-GS is a simple and reliable score for predicting outcome that may help in decision-making in the acute clinical practice, which is the most important stage that determines the fate of patients suffering CVT.

## Author contributions

AA, CC-B, EC, and MB developed the research protocol. MB and EC analyzed the data and prepared the first draft. EC, AA, FB, and CC-B reviewed and edited the first draft and final versions of the manuscript. All other authors provided data, reviewed results, provided guidance on methodology and data analysis or reviewed the manuscript, and approved the final version of the manuscript.

### Conflict of interest statement

MB has received research grants from Pfizer and Boehringer-Ingelheim. EC has received research grants from Sanofi; has served as a research adviser for Sanofi, the Ferrer Group, Novartis, TEVA, Eisai and Genzyme; and has received speaker honoraria from Novartis, Genzyme, and the Ferrer Group. FB has received speaker honoraria from Boehringer-Ingelheim and served as a research adviser for Boehringer-Ingelheim and Pfizer. CC-B has received research grants from Genzyme and Boehringer-Ingelheim; has served as a research adviser for Boehringer-Ingelheim, Bayer, and Pfizer; and has received speaker honoraria from Bayer, Boehringer-Ingelheim, and the Ferrer Group. AA has received speaker honoraria from Boehringer-Ingelheim and the Ferrer Group and served as a research adviser for Boehringer-Ingelheim, Bayer, and Pfizer. The remaining authors declare that the research was conducted in the absence of any commercial or financial relationships that could be construed as a potential conflict of interest.
